# Antifungal In Vitro Activity of Pilosulin- and Ponericin-Like Peptides from the Giant Ant *Dinoponera quadriceps* and Synergistic Effects with Antimycotic Drugs

**DOI:** 10.3390/antibiotics9060354

**Published:** 2020-06-23

**Authors:** Hilania Valéria Dodou Lima, Carolina Sidrim de Paula Cavalcante, Gandhi Rádis-Baptista

**Affiliations:** 1 Postgraduate Program in Pharmaceutical Sciences, Faculty of Pharmacy, Dentistry and Nursing, Federal University of Ceará, Fortaleza 60416-030, Brazil; valeriadodou@gmail.com; 2 Center for Science and Technology, State University of Ceará, Fortaleza 60714-903, Brazil; carolsidrim81@gmail.com; 3 Laboratory of Biochemistry and Biotechnology, Institute for Marine Sciences, Federal University of Ceara, Fortaleza 60416-030, Brazil

**Keywords:** *Dinoponera quadriceps*, giant ant venom-peptides, antimicrobial peptides, antifungal peptides, pilosulin-like peptides, ponericin-like peptides, fungicidal synergism, combination drugs, drug-resistant yeasts

## Abstract

Venoms from ants comprise a rich source of bioactive peptides, including antimicrobial peptides. From the proteome and peptidome of the giant ant *Dinoponera quadriceps* venom, members of five known classes of antimicrobial peptides were disclosed (e.g., dermaseptin-, defensin-, ICK-, pilosulin- and ponericin-like types). Based on comparative analysis, these family members have structural determinants that indicate they could display antimicrobial activities. In previous works, pilosulin- and ponericin-like peptides were demonstrated to be active against bacteria, fungi, and parasites. Herein, the antifungal activity of ponericin- and pilosulin-like peptides were assessed, aiming at the expansion of the knowledge about AMPs in predatory ants and the development of new microbicide strategies to deal with difficult-to-treat fungal infections. Synthetic pilosulin- (Dq-2562, Dq-1503, and Dq-1319) and ponericin-like (Dq-3162) peptides were evaluated for their fungicide and fungistatic activities against different species of *Candida*, including a drug-resistant clinical strain. The MICs and MLCs were determined for all peptides individually and in combination with general antifungal drugs by the microdilution method. The time-kill kinetic curves were set up by means of a luminescent reagent, of which the light signal is proportional to the number of viable cells. The candicidal synergism observed by the combination of subinhibitory concentrations of peptides and general antimycotic drugs were quantified by the checkerboard test and fluorescent dye permeation assay. The influence of ergosterol on the antifungal activity was verified by supplementation of culture medium. The pilosulin- (Dq-2562 and Dq-1503) and ponericin-like (Dq-3162) were the most active peptides, displaying a broad spectrum of antifungal activity in vitro, with MICs in the range of 0.625 to 10 µM. The combination of peptides and conventional antimycotic drugs displayed a synergistic reduction in the MIC values of individual peptides and drugs, while soluble ergosterol in the culture medium increased the MICs. The fungicide and fungistatic activity of the individual peptides and peptides in combination with antimycotics were time-dependent with a rapid onset of action and long-lasting effect, which involved membrane disruption as an underlying mechanism of their action. Altogether, pilosulin- and ponericin-like peptides from the giant ant *D. quadriceps* venom display a broad-spectrum of candicidal activity, what allows their inclusion in the row of the antifungal peptides and gives support for further studies on the development of strategies to fight candidiasis.

## 1. Introduction

Invasive fungal diseases have been considered, in recent decades, a threat to public health, due to the difficult diagnosis and treatment and the high morbidity and mortality [1,2,3]. Yeasts of the genus *Candida* are implicated in this clinical problem. Strains of *Candida* can express several molecular and cellular factors that modulate their level of virulence and the severity of candidiasis infections, such as the ability to produce adhesins and form biofilms, express invasins (proteins associated with pathogens’ penetration into host cells) and secrete tissue damage-cytolytic proteins and hydrolytic enzymes. In addition, *Candida* can express phenotypes that endow them to thrive in stressful conditions (e.g., pH changes), avoid phagocytosis, as well as evade other defense mechanisms of the host immune system. Moreover, *Candida* yeasts are capable of making a transition in their form of growth, from planktonic to hyphae or biofilms [4,5,6,7]. *Candida* species often cause epithelial infections in mouth, skin, and vagina, but systemic infections in hospitalized individuals are the most cumbersome, as they can contribute to high mortality rates [2,8]. Individuals and patients that are immunocompromised and/or are making use of antibiotics to treat bacterial infections are also at increased risk of candidiasis. The species *C. albicans* is the main cause of candidiasis and is responsible for 37% of systemic infections caused by the genus *Candida*, followed by *C. glabrata* (27%), *C. parapsilosis* (14%), *C. tropicalis* (8%), and *C. krusei* (2%). In invasive infections caused by *C. albicans*, a mortality rate of about 40% have been reported, even in patients in regimen of drug therapy [3,9]. Five main classes of antifungals are currently available on the market, but only three classes are known to be effective for the treatment of severe systemic *Candida sp*. infections; these classes comprise the azoles, echinocandins, and amphotericin B (a polyene) [8,10,11,12]. These antimycotic drugs exert their antifungal effects by interfering primarily with the metabolism of ergosterol in yeasts’ cell membrane and glucan, in yeasts’ cell wall. However, as presently known, these antifungals have some disadvantages that preclude their widespread use, since they display relatively high toxicity, interact with other therapeutic drugs, require intravenous administration and have a high cost for prolonged treatment [10]. Moreover, the increasing incidence of *Candida* infections, associated with the emergence of strains that are resistant to general antifungals, as well as the emergence of drug-resistance by non-albicans strains of *Candida*, have demanded a steady research and development of new broad- (or specifically narrow-) spectrum antifungals that exert their effects by alternative mechanisms of action, combined with low toxicity [8,12,13].

In view of this scenario and demand, antimicrobial peptides (AMPs) have been widely investigated as promising substances for the development of new therapeutic alternatives to fight microbial infections. AMPs have a wide range of microbial targets, being active against bacteria, yeasts, and enveloped viruses, and by acting through distinct mechanisms, they reduce the chance of microorganisms develop resistance to drugs [14,15]. AMPs are gene-encoded polypeptide products, expressed in organisms of all phyla, ranging from bacteria to high eukaryotes, as a component of the host’s innate immunity against pathogens. AMPs are generally characterized by short sequences of amino acids, low molecular mass and positively charged structures, that adopt an amphiphilic helix topology. Moreover, despite these general characteristics, AMPs are diverse in structural elements and singularities, such as post-translational modifications of longer preproprecursors structural and stabilization by disulfide-bond(s), as reviewed elsewhere [16,17]. The number of AMP structures and intrinsic activities have steadily increased, characterized and documented, so reclassification and regrouping that rely on the basic topologies or the molecular targets of AMPs have been under focus. For instance, antifungal peptides (natural or synthetic) can be proposedly grouped, regarding their mechanisms of action, in peptides that are able to cross cell membranes, form pores, and interfere with membrane permeability or stability (by interfering with the synthesis of glucans or chitin); and, alternatively, grouped in peptides that interfere with cell membrane homeostasis, intercalate the lipid bilayers (without membrane translocation), and cause cell lysis [18]. The intracellular targets for antifungal peptides are also diverse, like ability to bind to DNA and RNA, inhibit protein synthesis, and induce apoptosis-mediated cell death, to mention few examples [18,19].

Despite thousands of AMP structures characterized so far and known to date from numerous animal sources, including animal venom [20,21], bioactive peptides from ant venom, in particular, are still poorly described, compared to their counterparts in the venom of snakes, spiders, and scorpions. The richness of peptide diversity in ants have been recently noticed and a considerable number of important biological activities listed [22,23]. The advances in the disclosure and characterization of venom bioactive peptides from ants are granted by the refined and robust omics technologies currently available [24,25,26,27,28,29,30]. Among the venomous sting ants, *Dinoponera quadriceps* is one of the largest known predatory ants. The transcriptomic, proteomic, and peptidome analysis of *D. quadriceps* venom gland and crude venom revealed peptides that are structurally similar to insect defensins and other classes of linear peptides with predictable antimicrobial activities, such as temporin- and dermaseptin-like peptides, among others. In addition to potential AMPs, the venom cocktail of *D. quadriceps* is made up by neuroactive peptides, biogenic amines, allergens, and hydrolytic enzymes [25,31,32,33]. In a range of experiments for screening the antimicrobial potential of *D. quadriceps* venom components against representative strains of Gram-positive, Gram-negative bacteria and yeasts, pilosulin- and ponericin-like, collectively referred as dinoponeratoxins, were found to display broad spectrum of antimicrobial activity [32,33]. Indeed, previous studies with the crude venom and synthetic dinoponeratoxin-like peptides from *D. quadriceps* venom were reported to exhibit both antimicrobial and antiparasitic effects [34,35,36]. Antimicrobial activities of crude venom and purified venom-peptides from ants were also observed in congeneric and related ponerine ants [37,38,39]. Thus, peptides derived from the venom of ants appear as promising candidates for further investigation and characterization of potent antimicrobial peptides. In the present work, the antifungal activity of synthetic pilosulin- and ponericin-like peptides derived from sequences found in the venom of *D. quadriceps* were evaluated individually and in combination with general antimycotic drugs used in clinical settings against several *Candida* species and strains. The fungicide and fungistatic activity were time-dependent with a rapid onset of action and long-lasting effect. The candicidal activity of *D. quadriceps* peptides in synergistic combinations with general antimycotics, such as amphotericin B, displayed better performance, acting similarly as the individual peptides by cell membrane perturbation, but with negligible hemolytic effect in vitro.

## 2. Results

### 2.1. Peptides

The synthetic pilosulin- and ponericin-like peptides derived from D. quadriceps venom studied in this work are represented in Table 1. Accordingly, the synthetic peptides are linear, short sequence peptides identical to parental venom-derived peptides, which are the products of post-translational modification that released encrypted peptides from a longer preproprecursor, as is the case of pilosulin-like peptides. Synthetic ponericin-like peptide (Dq-3162) is identical to the natural post-translationally amidated-peptide found in the venom. Both pilosulin- and ponericin-like peptides virtually adopt alpha-helical topological structures, as seen by predicted models, with localized hydrophobic faces (Dq-2562 and Dq-3162) and values of hydrophobic moment (µ_H_) that are indicative of high degree of helicity and amphiphaticity with membrane-seeking properties, affinity for lipid membranes and propensity to form pores (higher H values and lower µ_H_ values). All peptides have net positive charges, ranging from +2 to +5 (Table 1). 

### 2.2. Evaluation of Antifungal Activity of Synthetic Pilosulin- and Ponericin-Like Peptides from D. quadriceps Venom

#### 2.2.1. Minimum Inhibitory Concentration (MIC) and Minimum Lethal Concentration (MLC)

The synthetic pilosulin- (Dq-2562, Dq-1503, and Dq-1319) and ponericin-like (Dq-3162) peptides presented a broad-spectrum of antifungal activity *in vitro*, with fungistatic and fungicidal effects against different species and strains of *Candida,* including a clinical strain resistant to amphotericin B and fluconazole. The most active peptide with the lowest MIC and MLC was Dq-3162. The only peptide that showed antifungal activity restricted to only two strains and with only fungistatic action was Dq-1319 (Table 2).

#### 2.2.2. Time-Dependent Antifungal Activity of *D. quadriceps* Peptides

**Figure 1:**
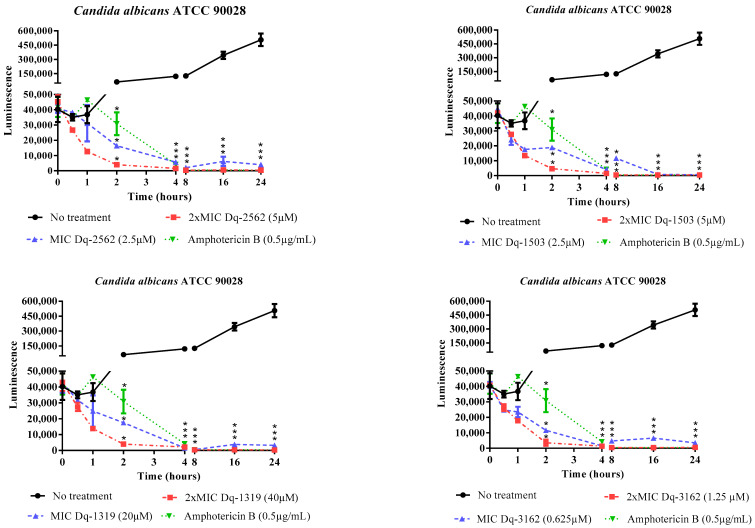
Effect of exposure time to synthetic pilosulin- (Dq-2562, Dq-1503, and Dq-1319) and ponericin-like (Dq-3162) peptides on the cell viability of *Candida albicans* ATCC 90028. ANOVA and Tukey’s multiple comparisons test. * *p* < 0.05 (compared to untreated *Candida* cells).

**Figure 2:**
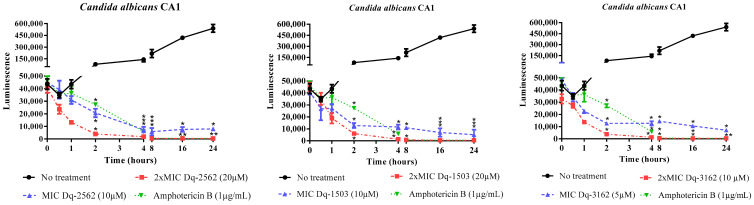
Effect of exposure time to synthetic pilosulin- (Dq-2562 and Dq-1503) and ponericin-like (Dq-3162) peptides from the giant ant *D. quadriceps* venom on the cell viability of amphotericin B- and fluconazole-resistant *Candida albicans* CA1. ANOVA and Tukey’s multiple comparisons test. * *p* < 0.05 (compared to untreated *Candida* cells).

The fungistatic and fungicidal effects of *D. quadriceps* peptides were demonstrated through the microbial growth and time-kill kinetics assay using the luminescent BacTiter-Glo® reagent, which reinforced the data obtained from the determination of the MICs and MLCs of all peptides. The onset of peptide action was as fast as 30 min, in all treatments, as measured by the reduction in the luminescent signal and, consequently, in the reduction of microbial cell viability. After 2 h of exposure, the reduction in microbial cell viability, in all treatments, showed statistically significant differences, compared to untreated *Candida* cells. The antifungal activity of all peptides was sustained up to 24 h of exposure and incubation. The peptides were active against both strains of susceptible and drug-resistant yeasts, and no difference in the pattern of growth kinetics and microbial death were observed between the sensitive (C. albicans ATCC 90028) and amphotericin B- and fluconazole-resistant *Candida* (*C. albicans* CA1), treated with the peptides Dq-3162, Dq-2562 and Dq-1503 (Figure 1, Figure 2).

### 2.3. Antifungal Activity of D. quadriceps Peptides in Combination with Antimycotyc Drugs 

#### 2.3.1. Checkerboard Test

The checkerboard test demonstrated that all peptides were able to interact synergistically with antimycotics used in clinical settings against most *Candida* strains. The combinations prepared with the peptides Dq-3162, Dq-2562, Dq-1503, and Dq-1319 and antimycotic drugs showed synergistic effects in the order of 60%, 52%, 64%, and 40%, respectively. It is important to note that, for the reference strains (*C. albicans* ATCC 90028), all combinations of these peptides with amphotericin B and nystatin were also synergic, with a reduction in the MICs of these drugs up to eight- and sixteen-fold, respectively. For the amphotericin B- and fluconazole-resistant *C. albicans* CA1 strain, only the interactions with amphotericin B were synergistic, with a four-fold reduction in the MIC of this drug. For the drug-resistant strain *C. krusei* ATCC 40095, which also has an intrinsic resistance to fluconazole, the peptide Dq-1503 was able to potentiate the action of fluconazole, reducing its MIC by sixteen-fold (Table 3).

#### 2.3.2. Time-Kill Effect of the Combinations of *D. quadriceps* Antimicrobial Peptides and Amphotericin B on the *Candida* Cell Viability

**Figure 3:**
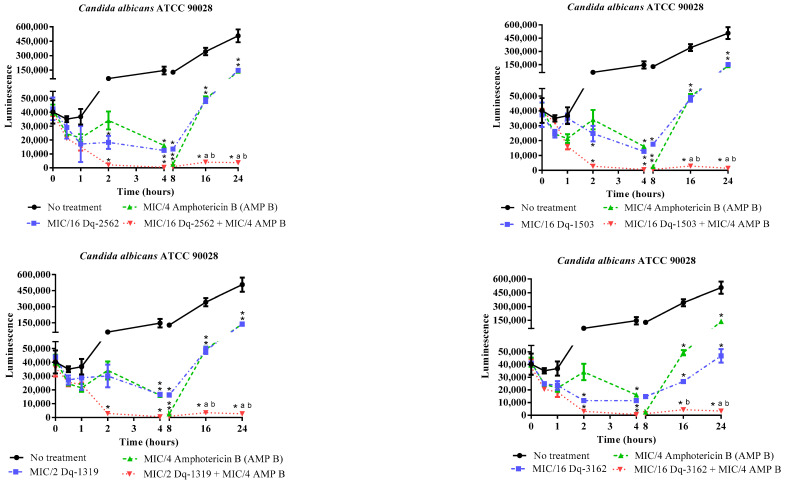
Time-kill effect of *D. quadriceps* antimicrobial peptides in combinations with amphotericin B on the cell viability of *Candida albicans* ATCC 90028. ANOVA and Tukey’s multiple comparisons test. * *p* < 0.05 (compared to untreated *Candida* cells). ^a^: *p* < 0.05 (compared to subinhibitory concentrations of individual peptides alone). ^b^ *p* < 0.05 (compared to subinhibitory concentration of amphotericin B, AMP B).

**Figure 4:**
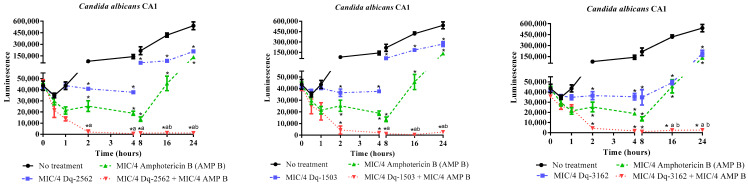
Time-kill effect of *D. quadriceps* antimicrobial peptides in combination with amphotericin B on the cell viability of *Candida albicans* CA1. ANOVA and Tukey’s multiple comparisons test. * *p* < 0.05 (compared to no treatment). ^a^ *p* < 0.05 (compared to subinhibitory concentration of individual peptides alone). ^b^ *p* < 0.05 (compared to subinhibitory concentration of amphotericin B, AMP B).

The synergism observed with combinations of peptides and conventional antimycotic drugs was also evaluated by microbial growth and time-kill kinetics using the luminescent BacTiter-Glo® reagent. The combinations that included individual peptides and amphotericin B were selected for this analysis. The time-kill curves obtained in this test series showed similar patterns observed in the kinetics’ test for individual peptides alone, i.e., the peptide and antimycotic combinations also reduced the cell viability of both susceptible and resistant strains, in the first 30 min of exposure. The contrastive differences between treated and untreated *Candida* cells were significant in a time course of 2 h of exposure. Between 2 h and 24 h of treatment, the combination of the four peptides individually and amphotericin B reduced the cell viability of both susceptible and resistant strains, to a minimum of cell viability, close to the detection limit of the assay, indicating a long-lasting fungicidal effect (Figure 3, Figure 4).

In Figure 3, the time-kill curves show the antifungal activity of the synthetic peptides in combination with amphotericin B against *C. albicans* ATCC 90028, at their subinhibitory concentrations. As can be seen, the four individual peptides in combination with amphotericin B are able to reduce the microbial viability within 8 h of exposure, even at concentrations that are 16-fold (for Dq-3162, Dq-2562, and Dq-1503) and 2-fold (for Dq-1319) lower than the MICs for the peptides alone. As noticed in Figure 4, after 4 h (for Dq-2562 and Dq-1503) or 8 h (for Dq-3162), at sub-MIC concentrations, the activity of peptides that were not associated with amphotericin B against *C. albicans* CA1, was still fungistatic. The fungistatic effect is also observed with amphotericin B at sub-MIC concentrations within the same time-frame.

#### 2.3.3. Membrane Permeabilization Induced by Pilosulin- and Ponericin-like Peptides

According to data from Table 4 and Figure S1, on the accumulation of the fluorescent dye SYTOX® Green in microbial cells with disrupted cytoplasmic membranes, the most active peptides pilosulin- (Dq-2562) and ponericin-like (Dq-3162) peptides, at their MICs, caused an increase in the cell number that stained positive for this cell impermeant nucleic acid stain, as consequence of the increased number of cells with compromised cytoplasmic membranes (54 to 76%, in 4 h of exposure). The synergistic combinations of peptides and amphotericin B, at their sub-MICs, caused similar effect of compromising *Candida* cell membranes and cell death (50 to 73%). Untreated *Candida* cells showed low uptake of SYTOX® Green, reflecting the low percentage of dead cells (4 to 5%). The average cell size of *Candida* cells treated with either individual peptides, amphotericin B or the combinations of peptides and amphotericin B, was reduced by up to 50% for both standard and drug-resistant clinical isolate strains in comparison to untreated cells (Table S1).

### 2.4. Hemolytic Activity of D. quadriceps Antimicrobial Peptides Alone and in Combinations with Amphotericin B

**Figure 5:**
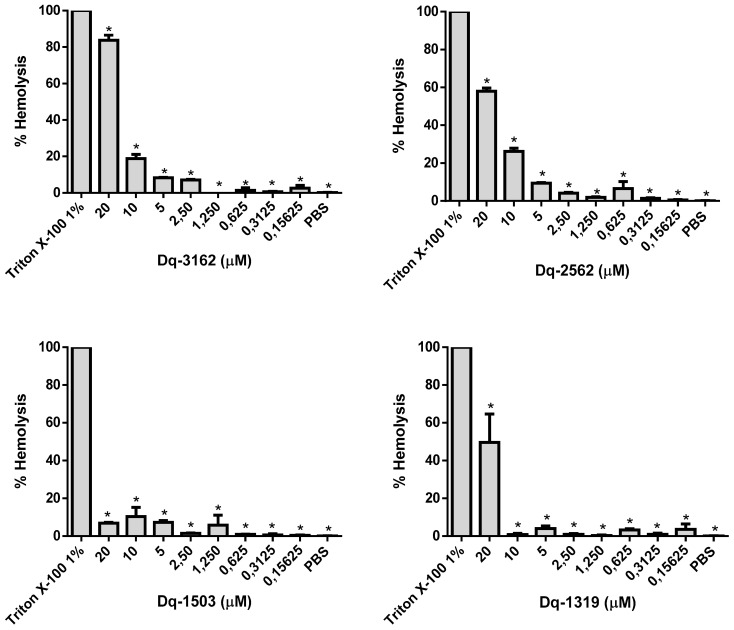
Percentage of hemolysis, in vitro, after exposure of human erythrocytes to synthetic pilosulin- (Dq-2562, Dq-1503, and Dq-1319) and ponericin-like (Dq-3162) peptides. Human red blood cells were treated with serial concentrations of these *D. quadriceps* antimicrobial peptides for 1 h, as described in materials and methods. ANOVA and Dunnett’s multiple comparisons test. * *p* < 0.05 (compared to 1% Triton X-100).

The MICs of Dq-3162 peptide for *Candida* sp. ranged from 0.0625 µM to 10 µM, and, in this concentration range, the percentage of hemolysis was between 1.3% and 18.7%. At highest concentration of Dq-3162 peptide, i.e., 20 µM, the hemolytic activity reached 83.7%. The Dq-2562 peptide exhibited hemolysis rates of 4.2% and 26.2% at the concentrations that exhibited antifungal activity (2.5 µM and 10 µM). At the concentration of 20 µM, this peptide was also highly cytotoxic to erythrocytes, causing 57.9% of hemolysis. The peptide Dq-1503 showed the lowest hemolytic effect, with percentage of hemolysis of 1.5% and 10.4%, at the concentrations of 2.5 µM and 20 µM. The peptide Dq-1319, in contrast, showed the highest hemolytic effect (49.5%) at the concentration in which its antifungal activity was observed (20 µM). At concentrations inferior to 2.5 µM, the hemolytic activity was effectively very low (< 1%) for all peptides tested (Figure 5).

**Figure 6:**
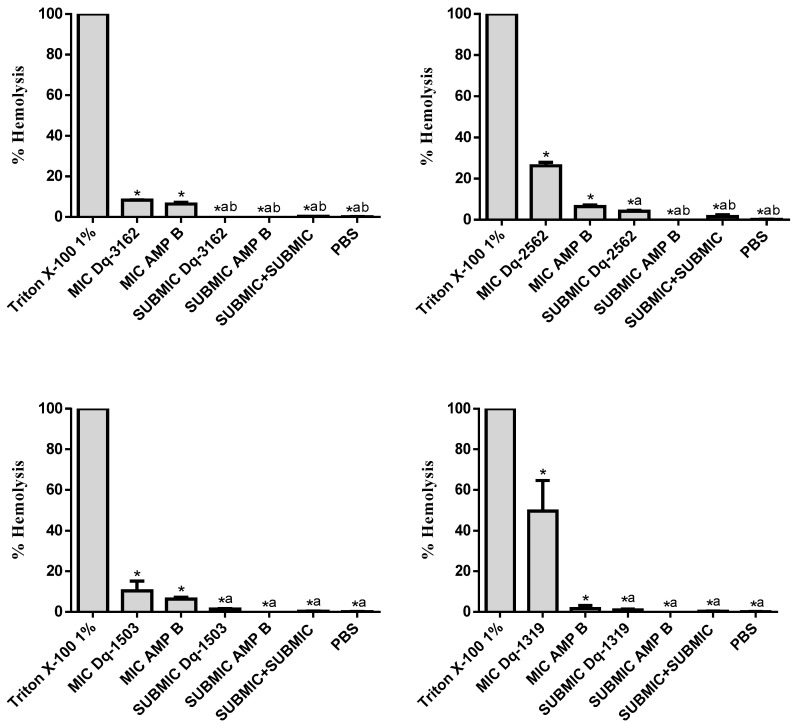
Percentage of hemolysis, *in vitro*, after exposure of human erythrocytes to combinations of pilosulin- (Dq-2562, Dq-1503, and Dq-1319) and ponericin-like (Dq-3162) peptides and amphotericin B. Human erythrocytes were treated as described in materials and methods. ANOVA and Tukey’s multiple comparisons test. * *p* < 0.05 (compared to 1% Triton X-100). ^a^ *p* < 0.05 (compared to the MICs of peptides). ^b^ *p* < 0.05 (compared to amphotericin B, AMP B, at the MIC).

In Figure 6, the percentage of hemolysis is shown for (1) the individual peptides and amphotericin B at their MIC-equivalent concentrations for the drug-resistant clinical strain *C. albicans* CA1 (for Dq-3162, Dq-2562, and Dq-1503) and for the standard strain *C. albicans* ATCC 90028 (for Dq-1319); and (2) for the combinations of individual peptides and amphotericin B, at their subinhibitory MICs (SUBMIC), which are equivalent to those concentrations that synergistic effects were observed for the same strains used in the checkerboard test. As can be seen, the combinations of the individual peptides and amphotericin B, at their subinhibitory concentrations (sub-MICs), in which that peptide and antimycotic drug together are synergistically fungicide, hemolysis activity was negligible, in vitro. 

### 2.5. Effect of Soluble Ergosterol on the Antifungal Activity of Peptides 

The presence of increasing amount of soluble ergosterol in the culture medium altered proportionally the MICs of all peptides for the different species of *Candida* sp tested, from 1- to 2-order (Table 5). For instance, for the peptide Dq-3162, the increase of the MICs increased from 0.0625 to 10 μM, in the presence of 0 to 800 μg mL^−1^ of ergosterol, respectively. Similarly, the MIC of amphotericin B, used as a positive control, was affected by the presence of increasing concentrations of soluble ergosterol in the culture medium; the amphotericin MIC increased from 0.5 μM to 32 μM, at concentrations of soluble ergosterol of 0 and 800 μg mL^−1^, respectively, in the culture medium.

## 3. Discussion

The venom of hymenopterans (ants, bees, and wasps) and other arthropods (e.g., spiders, scorpions, and centipedes) have been recognized as a rich source of antimicrobial peptides among other types of biologically active peptides, with a vast diversity of structures and broad-spectrum of molecular and cellular targets [21,40,41]. The crude venom of the giant ant *Dinoponera quadriceps* was previously observed to display in vitro antiparasitic effect against *Leishmania amazonensis* and *Trypanosoma cruzi*, mediated by apoptosis and necrosis [35]. The antibacterial activity of *D. quadriceps* crude venom against *Staphylococcus aureus* was also reported and the underlined mechanism of antimicrobial action was suggested to involve the interference with cell membrane permeability [34]. Synthetic dinoponeratoxin-derived peptides that are related to ponericins and pilosulins from the *D. quadriceps* venom showed antiparasitic activity in vitro, which were also mediated by apoptosis, in *T. cruzi* [36]. In disk diffusion test, the potential antimicrobial activity of isolated *D. quadriceps* peptides was observed in Gram-positive and Gram-negative bacteria, dermatophyte fungi and yeasts [32]. Recently, we have described five families of peptides present in the *D. quadriceps* venom peptidome and characterized the mast cell-degranulating, hemolytic, and antimicrobial activities of representative synthetic dinoponeratoxin-related peptides [33]. In that work, it was verified that such pilosulin- (Dq-2562) and ponericin-like (Dq-3162) peptides were active against bacteria and yeasts. Based on these previous reports, herein, the antifungal activity was forward investigated with the synthetic version of the linear, processed encrypted peptides, related to ponericin family of peptides, the peptides Dq-2562, Dq-1503, and Dq-1319, and the 28-residue ponericin-like peptide, Dq-3162. As above mentioned, revealed by the peptidome analysis of crude *D. quadriceps* venom, the peptides Dq-1319 and Dq-1503 are the processed post-translational peptide products of the parental Dq-2562, while Dq-3162 is the amidated peptide product of larger precursor of 30 amino acid residues [25,33]. Thus, post-translational modification operates in the venom gland of the giant ant *D. quadriceps* to increase the diversity and stability of peptide structures and, supposedly, to augment the range of biological activities in the venom. 

Regarding the antifungal effects of pilosulin- (Dq-2562, Dq-1503, and Dq-1319) and ponericin-like (Dq-3162) peptides of *D. quadriceps*, the results from the determination of the MICs and MLCs (Table 2) are corroborated by the time-kill kinetics (Figure 1 and Figure 2) and pointed out that the peptides Dq-3162, Dq-2562, and Dq-1503 display the most efficient fungicide activity. In general, membranolytic antimicrobial peptides (AMPs) are characterized by their short size, presence of hydrophobic and polar sides (i.e., amphipathicity), as well as helicity [42], and, indeed, these *D. quadriceps* peptides display most of these structural determinants. However as aforementioned, the topological structures and mechanisms of antimicrobial activity are numerous [17]. The same is valid for the antifungal peptides [18]. With exception of Dq-3162 (a ponericin-related), the pilosulin-like peptides Dq-2562, Dq-1503, and Dq-1319 are largely hydrophobic (Table 1) with the highest values of µH (hydrophobic moment). All peptides possess net positive charges. It is known that amphipathicity, cationicity, hydrophobicity, and charge distribution are important attributes for membrane interaction, membrane insertion, membranolysis, antimicrobial activity, and the level of cytotoxicity (e.g., hemolysis) [43,44]. As the first step of microbial killing, positively charged peptide usually interact with the pathogen membrane by electrostatic attraction via negatively charged components on bacterial (or yeast) plasma membrane. This is followed by peptide-membrane insertion, destabilization, and, eventually, pore formation, lysis, and cytoplasmic extravasation [45,46]. In concordance with these overall structural features and physicochemical properties, the peptide Dq-3162 and Dq-2562 displayed a considerable candicidal activity (Table 2) and proportionally better efficacy/cytotoxicity ratios. Such efficacy/cytotoxicity ratios are improved by the combinations of individual peptides and antimycotic drugs (Table 3). Indeed, by using a cell membrane impermeant, molecular probe that is specific for staining nucleic acids (SYTOX® Green), which permeate only cells with compromised cytoplasmic membranes, it was verified that membrane disruption is involved in the mechanism of action of these *D. quadriceps* ponericin- and pilosulin-like peptides alone, as well as in combinations with amphotericin B (Figure S1). Notably, once exposed to *D. quadriceps* antifungal peptides, the average size of *Candida* cells that were treated with either the individual peptides or the peptides in combinations with amphotericin B, was substantially reduced, as determined by cell size (contour) analysis in the Countess™ II FL Automated Cell Counter. This observation suggests a level of shrinkage of *Candida* cells that were exposed to peptides and a membrane disruption effect mediated by the peptides and their combinations (Table S1). The reduction of cell size and cell shrinkage that result from membrane permeability have been observed for other antimicrobial peptides that act by disrupting membrane integrity of microbes, such as Bac8c —a synthetic cationic peptide derived from bactenecin, found in bovine neutrophils [47]. Moreover, as demonstrated by the time-kill kinetics with a luminescent reagent, the pilolusin- and ponericin-like peptides, as seen here, displayed a rapid onset of fungicide action (~30 min) and a long-lasting (up to 24 h) antifungal effect in vitro (Figure 1 and Figure 2). 

Despite the appreciable antifungal activity of these peptides, a possible problem with all antimicrobial peptides for future development as therapeutic alternatives against microbial infection is their inherent cytotoxicity, like exemplified by hemolysis. This is particularly true whether the concentrations required to kill or inhibit the microbial growth are closed to the concentrations that provoke (cyto-)toxicity. In fact, venom and venom-peptides from ants and insects commonly cause cell lysis, like hemolysis, and tissue damage that are intrinsic biological properties that contribute to the overall envenomation symptoms [48,49]. Indeed, a number of AMPs are active due to their membranolytic mechanism of action [50,51], therefore these peptides are invariably and potentially cytotoxic whether they are not selective to the pathogen targets. Thus, thinking about the potential use of AMPs from venom, two immediate practical concepts could be envisioned and solutions carried out: One is the reduction of the potential cytotoxicity by amino acid substitution in a peptide-design program [52,53]. The second, more straightforward, is the reduction in the concentration of cytotoxic compounds through additive and, synergistic combinations and formulations, including combination of peptides and drugs. This later maneuver makes possible not only the maintenance of the therapeutic potency exerted by the individual substances in the drug combinations, but also makes some improvements due to the use of lower concentrations of the active substances. Moreover, the combination of drugs, including peptides, with distinct mechanisms of action is granted for fighting the emergence of drug resistance in pathogenic microbes. Thus, the search for synergistic combinations of AMPs and general antibiotics used in clinical settings are advantageous in decreasing the toxicity toward nontargeted healthy cells and restoring the susceptibility of microbes to the current drugs in the arsenal of antifungal chemotherapeutics [8,54,55]. In fact, examples of the combination of AMPs and general antifungals (e.g., amphotericin B and fluconazole) have been reported, as exemplified by β-peptide, crotalicidin-derived, and thionin-like peptides [56,57,58,59]. 

Herein, the pilosulin- (Dq-2562, Dq-1503, and Dq-1319) and ponericin-like (Dq-3162) peptides were all able to act synergistically with amphotericin B, by reducing their respective MICs and restoring the susceptibility of drug-resistant strains of *Candida* (Table 3). The synergistic effect was evidenced, by the checkerboard test, through the combination of peptides with the polyenic antifungals (nystatin and amphotericin B), which act by binding to ergosterol and, consequently, by altering the membrane permeability of yeasts [55]. The interaction of peptides with amphotericin B against *C. albicans* is interesting, seen that such an antifungal compound is a drug-of-choice in the treatment of systemic fungal infections, despite causing nephrotoxicity [8,55]. For this reason, the time-kill kinetics of the combinations of peptides and antimycotics were assessed with amphotericin B against strains of *C. albicans*. This kind of assessment indicated that fungicide activity, through the exposure to peptides and amphotericin B combinations (Figure 3 and Figure 4), displayed, similarly, a rapid onset of action and prolonged fungicidal effect, as observed for all individual peptides. Furthermore, the peptide-amphotericin B combinations, at their sub-MICs, preserved the effectiveness to kill *Candida* cell mediated by membrane-disruption (Table 4), but also reduced substantially the in vitro cytotoxicity as ascertained by the hemolysis assay (Figure 5 and Figure 6). Thus, the combination of existing therapies with adjuvant substances that improve antifungal activity, reduce the toxicity of the therapeutics, restore the MICs of resistant strains to the MICs of susceptible microbes, could contribute to overcome the resistance mechanisms of microorganisms to drugs. Moreover, they prolong the shelf life of current antifungals and, therefore, they are highly advantageous and should be further explored [54]. The analysis of the time-kill kinetic curves in Figure 3 and Figure 4 also call the attention to the effect exerted by the peptides alone, in subinhibitory concentrations. Although the candicidal effect was not total within 24 h, the subinhibitory concentrations of the peptides were sufficient to inhibit the yeast growth and/or to reduce the microbial cell viability for a considerable period of time (for 4 to 8 h). In addition, at these subinhibitory concentrations, the peptides did not show an appreciable in vitro cytotoxicity to red blood cells. These experimental evidences give support for further studies and usage of the combination of peptides and drugs at concentrations inferior to their MICs [60].

Finally, as above mentioned and experimentally confirmed for *D. quadriceps* peptides, by a fluorescent dye permeation assay with SYTOX® Green, membrane-targeting and permeabilization are intrinsic properties of most AMPs to exert their biological effect. The action on pathogen membranes can be accompanied of either membrane lysis and cytoplasmic extravasation or intracellular penetration and interaction with intracellular targets, among other particular mechanisms of action. Several reports have detailed the action of AMPs on the cell membranes of microorganisms [18,50,51,61,62] and pilosulin- and ponericin-like peptides from *D. quadriceps* could be added to these groups of membranolytic peptides. Thus, since the cell membrane of yeasts is mostly made up of ergosterol, it was important to investigate the influence of soluble ergosterol in the culture medium, on the activity of peptides, as the primary approach. Here, it was evidenced that the presence of soluble ergosterol in the culture medium caused changes in the MICs of all peptides, as well as the MIC of the polyenic antifungal amphotericin B, in a concentration-dependent fashion (Table 5). Thus, it is speculated the peptides might interact with or be sequestered by the soluble ergosterol in the culture medium, instead of binding directly to the membrane ergosterol on the surface of yeast membrane, consequently, decreasing the peptide to cell ratio and requiring higher concentrations of peptides to produce the antifungal effect. The influence of ergosterol in the activity of antifungal substances and extracts were previously observed by others [63,64]. In fact, the metabolism of ergosterol is the target for most clinically relevant antifungals and this sterol might have other biological roles in fungi than a structural participation in the composition of membranes [65]. Further, biophysical experiments should be conduct to investigate the peptide and lipid interaction with the aim of deep understanding the membranolytic mechanism of action of *D. quadriceps* peptides.

Altogether, the linear, amphiphilic pilosulin- (Dq-2562, Dq-1503, and Dq-1319), and ponericin-like (Dq-3162) peptides perform an efficient antifungal effect against susceptible and azole-resistant clinical strain of *Candida albicans*. Moreover, in combination with general antimycotic compounds (e.g., amphotericin) the cytotoxicity is reduced to a negligible level. In such peptide and antifungal drug combinations, the effective MICs for individual peptides and antifungal drug combined are decreased to sub-MICs, restoring the amphotericin B MIC to values found for susceptible strains of *Candida*. Moreover, the membrane perturbation mediated by *D. quadriceps* peptides appears as an important underlying mechanism of *Candida* cell death, in reason of the direct increase of membrane permeation to fluorescent dye and reduction of average cell size and cell shrinkage, as well as indirectly by the influence of soluble ergosterol on the antifungal activity of peptides. Further studies are invaluable to investigate the detailed mechanisms of peptide interaction with biological membranes and their components, as well as with other cellular and molecular targets. Last but not least, the formulation of *D. quadriceps* peptides and antimicrobial chemotherapeutics in combinations would be a valuable strategy for future research.

## 4. Materials and Methods 

### 4.1. Pilosulin- and Ponericin-Like Peptides from the Giant Ant D. quadriceps

The pilosulin-like peptides, Dq-2562, Dq-1503, and Dq-1319, were commercially prepared by solid phase peptide synthesis (China Peptides Co. Ltd., Shangai, China), with a minimum purity grade of 95%, as ascertained by HPLC and mass spectrometry analysis. The ponericin-like peptide Dq-3162 was also synthesized commercially (GeneScript Bioech Corp., Piscataway, NJ, USA), but it was a kind gift from Dr. K. Konno, the Institute of Natural Medicine, University of Toyama, Toyama, Japan. The peptides were weighed, solubilized in sterile deionized water to obtain a 1 mM stock solution, and stored in aliquots at –20 °C, until use.

### 4.2. Evaluation of Antifungal Activity of Peptides

#### 4.2.1. Microorganisms

The microorganisms used here were obtained from the Collection of Reference Microorganisms in Health Surveillance of the National Institute for Quality Control in Health, of the Oswaldo Cruz Foundation (CMRVS, FIOCRUZ-INCQS, Rio de Janeiro, Brazil). The amphotericin B- and fluconazole-resistant strain *Candida albicans* CA1 was obtained from a regional clinical laboratory.

#### 4.2.2. Determination of Minimum Inhibitory Concentration (MIC) and Minimum Lethal Concentration (MLC)

The determination of the MICs was as recommended by the Clinical and Laboratory Standards Institute in the reference method M27-A3 [66]. In order to prepare the inoculum, the *Candida* strains were cultured in Sabouraud broth, at 35 °C, for 24 h. Aliquots of these cultures were transferred to sterile test tubes containing 0.85% saline in order to obtain a turbidity equivalent to 0.5 McFarland standard (~10^6^ CFU/mL). This suspension was diluted to obtain inoculum with 10^4^ CFU/mL, which was used in the analysis. For determination of the MIC, 100 µL of Sabouraud broth with serial dilution of peptides (from 20 µM to 0.009 µM) and 100 µL of the microbial inoculum were mixed together in the wells of sterile 96-well microplates. The microplates were incubated at 35 °C for 24 h. After this period, the microbial growth was inspected by eyes. The MIC was considered the lowest concentration of the substance capable of inhibiting the fungal growth. Control of medium sterility (culture medium only), growth control (microbial suspension in culture medium) and control with a known antifungal drug (e.g., microbial suspension in culture medium containing amphotericin B) were included in the MIC determination. To find the MLC, 5 µL-aliquots of peptide and microbial suspension mixtures from the individual wells, which did not show microbial growth, in the MIC assay, were transferred to Petri-dishes containing Plate-Count agar and incubated at 35 °C for 24 h. Colonies were counted and the MLC was considered e the lowest concentration of peptide that caused, at least, 99.9% of cell death, compared to the inoculum used initially [67,68]. The MICs and MLCs of amphotericin B, miconazole, fluconazole, nystatin and cyclopyrox were also determined for all strains, according to the same methodology described above. The tests were performed in triplicate and the data were used to select the peptides and strains used in the subsequent tests.

#### 4.2.3. Determination of Time-Kill Kinetics of Peptide Activity Against *C. albicans*

This assay was performed with the strain *C. albicans* ATCC 90028 and the drug-resistant clinical strain *C. albicans* CA1, the pilosulin-like peptides Dq-2562 and Dq-1503, as well as the ponericin-like peptide, Dq-3162. To sterile microtubes, 100 µL of peptide solution, at concentrations equivalent to the MIC and 2 × MIC, were added and mixed with 100 µL of microbial inoculum (0.5 McFarland standard, 10^6^ CFU/mL). The microtubes were incubated at 35 °C and, in a time-frame of 0.0, 0.5, 1, 2, 4, 8, 16, and 24 h, and 50 µL were taken and transferred to 96-well flat bottom black microplates. Then, 50 µL of the BacTiter-Glo® reagent (Promega, Madison, WI, USA) was added to every well and the plates were incubated for 5 min, in the dark, under gentle agitation. Luminescence was measured with the Synergy HT multiple detection microplate reader (BioTek, Winoski, VT, USA). Untreated microbial suspensions and treated with a general antifungal drug (amphotericin B) were used as controls. Also, wells containing only Sabouraud broth were used to obtain background values of luminescence. The test was performed in triplicate.

### 4.3. Antifungal Activity of the Combinations of D. quadriceps Antimicrobial Peptides and Antimycotycs 

#### 4.3.1. Checkerboard Test

This test was performed with the strains *C. albicans* ATCC 90028, C. tropicalis ATCC 13803, *C. krusei* ATCC 40095, *C. parapsilosis* ATCC 40038, and *C. albicans* CA1. The reference antimycotic drugs used were amphotericin B, fluconazole, miconazole, cyclopyrox, and nystatin. The peptides were tested based on the results found in the determination of individual MICs. In 96-well microplates, 50 µL peptide solution in culture medium and 50 µL of individual antifungal were mixed and diluted in Sabouraud broth to reach final concentrations of ½, ¼, 1/8 and 1/16 × MIC. Then, 100 µL of microbial suspension (10^4^ CFU/mL) were transferred and homogenized by gently mixing. The microplates were incubated at 35 °C, for 24 h and microbial growth inspection by eyes. The test was performed in triplicate. Controls of medium sterility (culture medium only) and cell growth (microbial suspension in culture medium) were included [69]. To assess the antifungal activity of the combinations of *D. quadriceps* antimicrobial peptides and general antimycotics, the fractionated inhibitory concentration index (FICI) was calculated as follows:

FICI = (MIC of the peptide alone / MIC of the peptide in the combination) + 

(MIC of the antifungal alone / MIC of the antifungal in the combination)

The FICI was interpreted as: Synergistic effect, when FICI ≤ 0.5; additive effect, when FICI > 0.5 and < 1.0; and antagonistic effect, when FICI > 1.0 [70,71].

#### 4.3.2. Determination of the Time-Kill Kinetics of the Combination of *D. quadriceps* Antimicrobial Peptides and Amphotericin B on *Candida* Cell Viability

This assay was carried out with combinations of the four peptides individually and amphotericin B against strain *C. albicans* ATCC 90028, and with the pilosulin- (Dq-2562 and Dq-1503) and ponericin-like (Dq-3162) peptides against the amphotericin B- and fluconazole-resistant *C. albicans* CA1. Aliquots of 50 µL of peptides and 50 µL of the antifungal drug were mixed in microtubes, at their subinhibitory concentrations that showed the best results in the checkerboard assay. Afterward, 100 µL of microbial inoculum (0.5 McFarland standard, 10^6^ CFU/mL) were transferred and mixed The mixtures were incubated at 35 °C and, in a time-frame of 0.0, 0.5, 1, 2, 4, 8, 16, and 24 h of incubation, 50 µL aliquots were removed and transferred to individual wells in a 96-well flat bottom black microplates. Subsequently, 50 µL of the BacTiter-Glo® reagent (Promega, Madison, WI, USA) was added to each well and the plates were incubated for 5 min, in the dark, under gentle agitation. Luminescence was measured with the Synergy HT multiple detection microplate reader (BioTek, Winoski, VT, USA). Untreated microbial suspensions and treated only with peptides or only with the reference antifungal (amphotericin B) were used as controls. Wells containing only Sabouraud broth were used to obtain the background values of luminescence. The test was performed in triplicate

#### 4.3.3. Membrane Permeabilization Induced by pilosulin- (Dq-2562) and ponericin-like (Dq-3162) Peptides and Combinations with Amphotericin B

To check the membrane disruption effect caused by the most active peptides, Dq-2562 and Dq-3162, a cell impermeant fluorescent dye, SYTOX® Green nucleic acid stain (Molecular Probes, Eugene, OR, USA) was used. This dye penetrates only cells with damaged cytoplasmic membranes and is impermeable to live cells with intact membranes. In this assay, the two strains of Candida albicans were tested, as described for time-kill kinetics, in 4.2.3. Aliquots of 100 µL cell suspension of the strain *C. albicans* ATCC 90028 and the drug-resistant clinical strain *C. albicans* CA1, 0.5 McFarland standard (10^6^ CFU/mL), were incubated (35 °C, 4 h) with the peptides at their MICs and the sub-MICs when in combinations with amphotericin B for observation of their synergistic effect. After treatment, cells were collected by centrifugation (12,000× *g*, 4 °C, 2 min) and the culture medium removed and discarded. Cells were resuspended in the same original volume with 10 mM HEPES, 150 mM NaCl, pH 7.6, and SYTOX® Green (10 µM) added. After gently homogenization, the suspensions were incubated for 10 min and the dead cells, with disrupted membranes, counted with the Countess™ II FL Automated Cell Counter (ThermoFisher Scientific, Waltham, MA, EUA), in the GFP channel. Total cell number (live and dead cells) was enumerated in the brightfield. The contour (shape) analysis estimated the average Candida cell size of populations that were to the peptides and drug in comparison with untreated cells. The ratio of dead green/live cells was expressed in percentage. Untreated strain suspensions and treated with amphotericin B were used as controls. 

### 4.4. Hemolytic Assay

Fresh human blood from consented healthy donor was collected into vacutainer tubes (BD, Beckton Dickinson, São Paulo, Brazil) and centrifuged at 1000× *g*, for 10 min, at 4 °C. The plasma was removed and the pellet containing the red blood cells was rinsed three times with sterile PBS (35 mM phosphate, 150 mM NaCl, pH 7.4). After rinsing, cell pellet was resuspended in PBS to obtain an 8% (v/v) suspension of red blood cells. A 100 µL aliquot of this suspension was transferred to sterile microtubes containing 100 µL of individual peptide solutions (at concentrations ranging from 40 µM to 0.3125 µM). The final concentrations of red blood cells and peptides, in the hemolytic assay, were 4% (v/v) and 20 µM to 0.1562 µM of peptides, respectively. After incubation (37 °C, 60 min), under slight agitation, the microtubes were centrifuged at 1000× *g* for 2 min, the supernatants were collected and transferred to 96-well flat bottom clear plates and the quantity of hemoglobin released from lysed erythrocytes was estimated at 540 nm, with the Synergy HT multiple detection microplate reader (BioTek, Winoski, VT, USA). Untreated red cell suspensions and treated with 1% Triton X-100 were used as negative and positive controls, respectively [72]. The percentage of hemolysis was determined as:

(DO^540nm^ peptide and/or drug treated cells – DO^540nm^ untreated cells) / (DO^540nm^ Triton X-100 treated cells– DO^540nm^ untreated cells) × 100.

The hemolytic activity of some peptide and antifungal combinations was also assessed using the same protocol described above, except by using 50 µL of each individual substance used in this study. All tests were performed in triplicate.

### 4.5. Influence of Ergosterol on the MIC of Peptides and Amphotericin B 

The MICs of peptides for the strains *C. albicans* ATCC 90028, *C. tropicalis* ATCC 13803, *C. krusei* ATCC 40095, and *C. parapsilosis* ATCC 90038 were determined by the microdilution technique as described in 4.2.2. (reference method M27-A3, CLSI [66]), except that the medium was supplemented with increasing concentrations of ergosterol (100, 200, 400, and 800 µg/mL) in the culture medium. The assay was carried out in triplicate. Negative (culture medium) and positive growth controls (microbial suspension in culture medium) were included with amphotericin B used as a reference antifungal drug.

## 5. Conclusions

In the present work, it was evidenced that synthetic pilosulin (Dq-2562, Dq-1503, and Dq-1319) and ponericin-like (Dq-3162) peptides, identical to the parental peptides found in the venom of *D. quadriceps* giant ant, display a broad-spectrum of antifungal activity against all species of *Candida* sp. tested. With fungistatic and fungicidal effects, these *D. quadriceps* venom-derived peptides possess a rapid onset of action and long-lasting antifungal effect. In addition, these peptides are able to synergistically modulate the action of general antifungal drugs of different classes, as exemplified here. Although the individual peptides caused certain level of *in vitro* cytotoxicity to human red blood cells, at concentrations equivalent to their MICs, this was not the case when in combinations with amphotericin B, at their sub-MICs, which ensured the antifungal effect at such very low concentrations, but with negligible hemolytic activity. The peptides alone, even at subinhibitory concentrations, are also sufficient to sustain the antifungal effect for 4 to 8 h, without appreciable hemolysis. Thus, the pilosulin- and ponericin-like peptides from the venom of *D. quadriceps*, alone or in combination with current antimicrobial drugs, were demonstrated here to be potentially promising for the development of effective alternatives against fungal infections. The fact of these peptides exerts their effect by disrupting the plasma membrane is an advantageous mechanistic feature to make difficult the development of drug-resistance in pathogenic yeasts. Further studies are required to understand the detailed mechanisms of membrane interaction and destabilization, as well as to search for additional cellular and molecular targets of these pilosulin- and ponericin-like peptides. Moreover, from the viewpoint of pharmaceutical sciences, the study of combination and formulation of *D. quadriceps* peptides and antimicrobial chemotherapeutics could be promising for the antimycotic adjuvant medication. 

## Figures and Tables

**Table 1:** Structures, predict models and physicochemical characteristics of synthetic pilosulin- (Dq-2562, Dq-1503, and Dq-1319) and ponericin-like (Dq-3162) peptides from the venom of giant ant *Dinoponera quadriceps*.

Peptide	Primary and Secondary Structures ^(a)^	Helical Wheel Plot ^(b)^	Physicochemical Properties ^(c,d)^
Dq-1319	**_1_FWGTLAKWALK_11_** 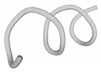	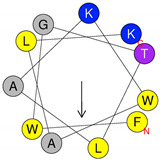	Exp. MW = 1320.75 Net charge = +2 H = 0.781 µ_H_ = 0.523 Hydrophobic face: none
Dq-1503	**_1_FWGTLAKWALKAI_13_** 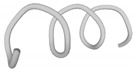	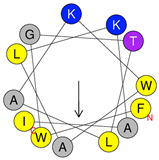	Exp. MW = 1504.86 Net charge = +2 H = 0.823 µ_H_ = 0.531 Hydrophobic face: none
**Dq-2562**	**_1_FWGTLAKWALKAIPAAMGMKQNK_23_** 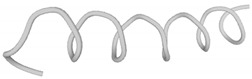	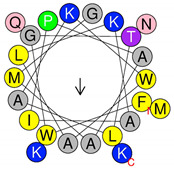	Exp. MW = 2561.38 Net charge = +4 H = 0.509 µ_H_ = 0.248 Hydrophobic face: IAML
**Dq-3162**	**_1_GLKDWWNKHKDKIVKVVKEMGKAGINAA_28_-NH_2_** 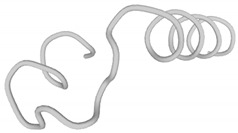	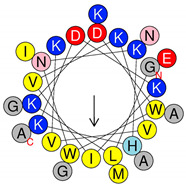	Exp. MW = 3162.75 Net charge = +5 H = 0.485 µ_H_ = 0.194 Hydrophobic face: LMIWGV

^a^ Secondary structural models were predicted with PepFold Server (https://bioserv.rpbs.univ-paris-diderot.fr/services/PEP-FOLD/). The chemical group representation (-NH_2_) means N-terminal amidated peptide. ^b^ Helical wheel projections and physicochemical properties were obtained by means of the “Heliquest” software (http://heliquest.ipmc.cnrs.fr/). ^c^ H, hydrophobicity; µ_H_, hydrophobic moment. The hydrophobic faces when present are indicated. The N- and C- terminal residues are indicated. ^d^ The experimental molecular weight (Exp. MW) were as reported in our previous work [33].

**Table 2:** Minimum inhibitory concentration (MIC) and minimum lethal concentration (MLC) of synthetic pilosulin- (Dq-2562, Dq-1503, and Dq-1319) and ponericin-like (Dq-3162) peptides from the giant ant *D. quadriceps* venom against *Candida* yeasts.

Strain	Conc.*	Dq-3162	Dq-2562	Dq-1503	Dq-1319
*Candida albicans* ATCC 90029	MIC MLC	10 (32) 20 (64)	10 (25) 10 (25)	- -	- -
*Candida albicans* ATCC 90028	MIC MLC	0.625 (2.0) 1.25 (4.0)	2.5 (6.25) 5.0 (12.5)	2.5 (3.75) 2.5 (3.75)	20 (26) -
*Candida tropicalis* ATCC 13803	MIC MLC	0.625 (2.0) 0.625 (2.0)	2.5 (6.25) 5.0 (12.5)	2.5 (3.75) 5.0 (7.5)	20 (26) -
*Candida tropicalis* ATCC 750	MIC MLC	10 (32) 20 (64)	10 (25) 20 (50)	- -	- -
*Candida parapsilosis* ATCC 90018	MIC MLC	10 (32) 10 (32)	10 (25) 10 (25)	20 (30) 20 (30)	- -
*Candida parapsilosis* ATCC 40038	MIC MLC	5.0 (16) 5.0 (16)	5.0 (12.5) 10 (12.5)	10 (15) 10 (15)	- -
*Candida krusei* ATCC 40095	MIC MLC	2.5 (8) 5.0 (16)	2.5 (6.25) 5.0 (12.5)	10 (15) 10 (15)	- -
*Candida krusei* ATCC 40147	MIC MLC	2.5 (8) 5.0 (16)	5.0 (12.5) 5.0 (12.5)	10 (15) 10 (15)	- -
*Candida albicans* CA1	MIC MLC	5.0 (16) 10 (32)	10 (25) 20 (50)	10 (15) 20 (30)	- -

Conc.*, concentration in µM and (µg mL^-1^); “-“: inactive (up to 20µM).

**Table 3:** Modulatory antifungal activity of synthetic pilosulin- (Dq-2562, Dq-1503, and Dq-1319) and ponericin-like (Dq-3162) peptides in combination with general antimycotics on the growth of pathogenic yeasts.

Peptide/ Antifungal	*C. albicans* ATCC 90028		*C. tropicalis* ATCC 13803		*C. krusei* ATCC 40095		*C. parapsilosis* ATCC 40038		*C. albicans* CA1	
	FICI	Reduction [ATF]	FICI	Reduction [ATF]	FICI	Reduction [ATF]	FICI	Reduction [ATF]	FICI	Reduction [ATF]
**Dq-3162/**										
AMP B	0.3125 (S)	4x	0.3125 (S)	4x	0.3125 (S)	4x	0.375 (S)	8x	0.5 (S)	4x
MICO	-	-	-	-	0.5 (S)	4x	0.3125 (S)	16x	0.5625 (A)	16x
CICL	0.3125 (S)	4x	0.5625 (A)	2x	0.3125 (S)	4x	0.3125 (S)	16x	0.5625 (A)	16x
FLUC	-	-	-	-	0.5625 (A)	16x	0.375 (S)	8x	0.5625 (A)	16x
NYST	0.1875 (S)	8x	0.1875 (S)	8x	0.1875 (S)	8x	0.1875 (S)	8x	0.5625 (A)	16x
**Dq-2562/**										
AMP B	0.3125 (S)	4x	0.3125 (S)	4x	0.3125 (S)	4x	0.3125 (S)	4x	0.5 (S)	4x
MICO	0.5625 (A)	16x	0.5625 (A)	16x	0.5625 (A)	16x	0.3125 (S)	16x	0.5625 (A)	16x
CICL	0.375 (S)	8x	0.5625 (A)	16x	0.3125 (S)	4x	0.3125 (S)	16x	0.5625 (A)	16x
FLUC	-	-	-	-	0.5625 (A)	2x	0.5625 (A)	16x	0.5625 (A)	16x
NYST	0.1875 (S)	8x	0.1875 (S)	8x	0.1875 (S)	8x	0.1875 (S)	8x	0.5625 (A)	16x
**Dq-1503/**										
AMP B	0.3125 (S)	4x	0.3125 (S)	4x	0.3125 (S)	4x	0.3125 (S)	4x	0.5 (S)	4x
MICO	-	-	-	-	0.3125 (S)	16x	0.375 (S)	8x	0.5625 (A)	16x
CICL	0.3125 (S)	4x	0.625 (A)	8x	0.3125 (S)	16x	0.3125 (S)	16x	0.5625 (A)	16x
FLUC	0.625 (A)	8x	0.625 (A)	8x	0.3125 (S)	16x	0.3125 (S)	16x	0.5625 (A)	16x
NYST	0.1875 (S)	8x	0.1875 (S)	8x	0.3125 (S)	16x	0.1875 (S)	8x	0.5625 (A)	16x
**Dq-1319**										
AMP B	0.75 (A)	4x	0.375 (S)	4x	ND		ND		ND	
MICO	0.5625 (A)	16x	0.5625 (A)	16x	ND		ND		ND	
CICL	0.5 (S)	4x	0.5625 (A)	16x	ND		ND		ND	
FLUC	0.5625 (A)	16x	0.5625 (A)	16x	ND		ND		ND	
NYST	0.1875 (S)	8x	0.1875 (S)	8x	ND		ND		ND	

AMP B (Amphotericin B), MICO (Miconazole), CICL (Ciclopirox), FLUC (Fluconazole), NYST (Nystatin), FICI (Fractional Inhibitory Concentration Index), Reduction (Reduction in antifungal concentration), ND (Not determined). (S): Synergistic effect. (A) Additive effect. Hyphen, “-“, means that there was no inhibition of microbial growth in the combinations of peptides and antimycotic drugs.

**Table 4:** Accumulation of SYTOX® Green in *Candida* cells with disrupted membranes induced by pilosulin- (Dq-2562), ponericin-like (Dq-3162) peptides and peptide combinations with amphotericin B.

Peptide/Drug ^a^	Concentration ^b^	SYTOX® Green Stained-Cells ^c^ (%)	
		*C. albicans* ATCC 90028	*C. albicans* CA1 ^(d)^
Dq-2562	MIC	75	56
Dq-3162	MIC	76	54
Amphotericin B	MIC	48	58
Dq-2562/AMP B	SUBMICs	73	69
Dq-3162/AMP B	SUBMICs	50	59
-	-	5	4

^a^*D. quadriceps* peptides and amphotericin B were used alone or in combinations; ^b^ concentrations were either at their respective MICs or at their sub-MICs when combinations of peptides and amphotericin B were tested; ^c^ percentage of membrane-permeated cells in relation to total cell number, which were enumerated after 4 h of treatment with either individual peptides, amphotericin B or the combinations of them; ^d^ clinical strain of antimycotic drug-resistant *C. albicans*. The hyphen “-“, untreated yeast cells.

**Table 5:** The effect of soluble ergosterol in the culture medium on the minimum inhibitory concentration (MIC) of pilosulin- (Dq-2562, Dq-1503, and Dq-1319) and ponericin-like (Dq-3162) peptides for pathogenic yeasts.

Peptide	*C. albicans* ATCC 90028	*C. tropicalis* ATCC 13803	*C. krusei* ATCC 40095	*C. parapsilosis* ATCC 40038
**Dq-3162**				
Without ergosterol	0.0625 (2)	0.0625 (2)	2.5 (8)	5.0 (16)
With ergosterol (µg/mL)				
100	1.25 (4)	1.25 (4)	5.0 (16)	5.0 (16)
200	1.25 (4)	2.5 (8)	5.0 (16)	10 (32)
400	5.0 (16)	5.0 (16)	10 (32)	20 (64)
800	10 (32)	10 (32)	20 (64)	20 (64)
**Dq-2562**				
Without ergosterol	2.5 (6.25)	2.5 (6.25)	2.5 (6.25)	5.0 (12.5)
With ergosterol (µg/mL)				
100	5.0 (12.5)	5.0 (12.5)	5.0 (12.5)	5.0 (12.5)
200	5.0 (12.5)	5.0 (12.5)	5.0 (12.5)	5.0 (12.5)
400	20 (50)	20 (50)	10 (25)	20 (25)
800	20 (50)	20 (50)	20 (50)	20 (25)
**Dq-1503**				
Without ergosterol	2.5 (3.75)	2.5 (3.75)	10 (15)	10 (15)
With ergosterol (µg/mL)				
100	2.5 (3.75)	5.0 (7.5)	10 (15)	10 (15)
200	5.0 (7.5)	5.0 (7.5)	10 (15)	20 (30)
400	10 (15)	10 (15)	20 (30)	20 (30)
800	20 (30)	10 (15)	20 (30)	40 (60)
**Dq-1319**				
Without ergosterol	20 (26)	20 (26)	-	-
With ergosterol (µg/mL)				
100	20 (26)	20 (26)	-	-
200	40 (52)	40 (52)	-	-
400	80 (104)	40 (52)	-	-
800	80 (104)	80 (104)	-	-
**Amphotericin B**				
Without ergosterol	0.5 (0.5)	0.5 (0.5)	1.0 (0.9)	0.5 (0.5)
With ergosterol (µg/mL)				
100	4.0 (3.7)	2.0 (1.8)	4.0 (3.7)	4.0 (3.7)
200	4.0 (7.4)	4.0 (3.7)	4.0 (3.7)	16 (15)
400	16 (15)	16 (15)	16 (15)	32 (30)
800	32 (30)	32 (30)	32 (30)	32 (30)

The hyphen denotes lack of activity (“-“: inactive). Concentration is in µM and (µg mL^−1^) for peptides and amphotericin B.

## Supplementary Materials

The following are available online at https://www.mdpi.com/2079-6382/9/6/354/s1, Figure S1: Accumulation of SYTOX® Green in cells with disrupted membranes provoked by pilosulin- and ponericin-like peptides, Table S1: Average cell size of Candida cells treated with pilosulin- (Dq-2562) and ponericin-like (Dq-3162) peptides, and peptide combinations with amphotericin B.
