# Supplementary material for: Antifungal In Vitro Activity of Pilosulin- and Ponericin-Like Peptides from the Giant Ant Dinoponera quadriceps and Synergistic Effects with Antimycotic Drugs
Source: Antibiotics (Basel). 2020 Jun 23;9(6):354. doi: 10.3390/antibiotics9060354 (PMC7344683; doi:10.3390/antibiotics9060354)

**Figure supplementary 1. Accumulation of SYTOX® Green in cells with disrupted membranes provoked by pilosulin- and ponericin-like peptides.** In images of bright field (odd numbers), live and dead cells of *Candida albicans* ATCC 90028 (A) and the drug-resistant clinical isolate *C. albicans* CA1 (B) are included. In the GFP channel (even numbers), membrane-damaged cells that accumulated the impermeant SYTOX® Green fluorescent dye are visible. Yeast cells were exposed to the peptide MICs, at 35 °C for 4h (A3 and A4, B3 and B4, Dq-3162; A5 and A6, B5 and B6, Dq-2562). Untreated cells at the same incubation conditions served as controls (A-1 and A-2, B1 and B-2). Cells were collected, washed and resuspended in 10 mM HEPES, 150 mM NaCl, pH 7.6. SYTOX® Green (10 µM) was added to cell suspensions, homogenized and incubated for 10 min. Cells were analyzed with the Countess II FL Automated Cell Counter (ThermoFisher Scientific, Waltham, MA, EUA). Green fluorescence indicates dead cells with permeabilized cytoplasmic membranes.


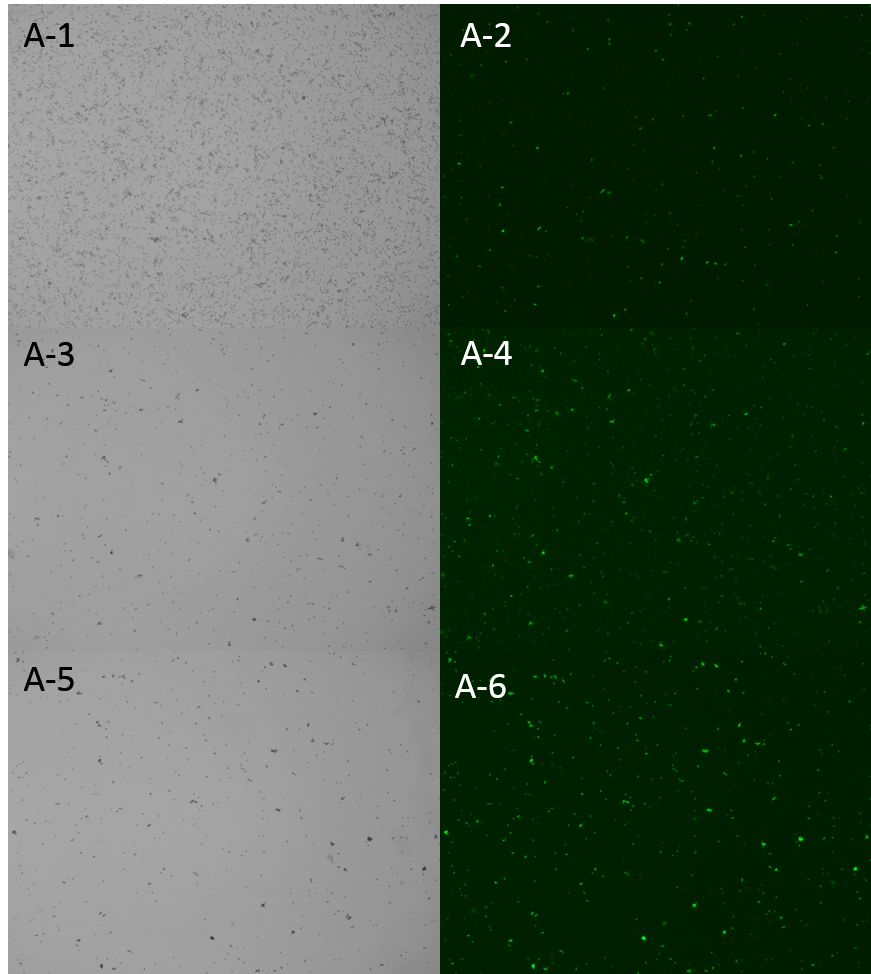


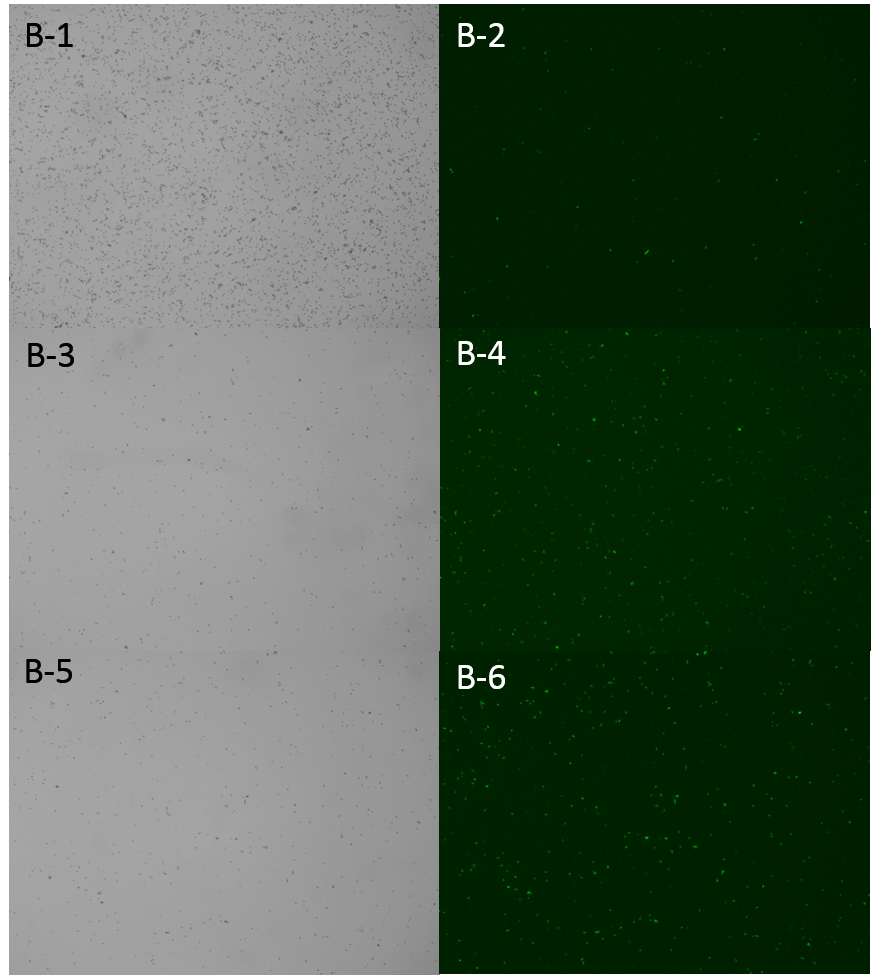

Supplement: Supplementary file 1 [file antibiotics-09-00354-s001.zip › Supplementary material/Supplementary Figure 1.docx]
