# Supplementary material for: Antifungal In Vitro Activity of Pilosulin- and Ponericin-Like Peptides from the Giant Ant Dinoponera quadriceps and Synergistic Effects with Antimycotic Drugs
Source: Antibiotics (Basel). 2020 Jun 23;9(6):354. doi: 10.3390/antibiotics9060354 (PMC7344683; doi:10.3390/antibiotics9060354)
Supplement: Supplementary file 1 [file antibiotics-09-00354-s001.zip › Supplementary material/Supplementary Table 1.docx]

Supplementary Table 1. Average cell size of *Candida* cells treated with pilosulin- (Dq-2562) and ponericin-like (Dq-3162) peptides, and peptide combinations with amphotericin B

| Peptide/drug^a^ | Concentration^b^ | Average cell size  (μM)^c^ | |
| --- | --- | --- | --- |
|  |  | *C. albicans* ATCC 90028 | *C. albicans* CA1 |
| Dq-2562 | MIC | 3,18 | 3.03 |
| Dq-3162 | MIC | 2.54 | 2.76 |
| Amphotericin B | MIC | 3,89 | 3.70 |
| Dq-2562/AMP B | SUBMICs | 2.46 | 2.96 |
| Dq-3162/AMP B | SUBMICs | 3.96 | 3.46 |
| - | - | 6.03 | 5.85 |

^a^*D. quadriceps* peptides and amphotericin B was used alone or in combinations; ^b^ concentrations were either at their respective MICs or at their sub-MICs, when in combinations of peptides and amphotericin B were tested; ^c^as determined by using the Countess™ II FL Automated Cell Counter (ThermoFisher Scientific, Waltham, MA, EUA). The hyphen “-“, untreated yeast cells.
